# End-to-End Learning Framework for IMU-Based 6-DOF Odometry

**DOI:** 10.3390/s19173777

**Published:** 2019-08-31

**Authors:** João Paulo Silva do Monte Lima, Hideaki Uchiyama, Rin-ichiro Taniguchi

**Affiliations:** 1 Departamento de Computação, Universidade Federal Rural de Pernambuco, Recife 52171-900, Brazil; 2 Voxar Labs, Centro de Informática, Universidade Federal de Pernambuco, Recife 50740-560, Brazil; 3 Library, Kyushu University, Fukuoka 819-0395, Japan; 4 Faculty of Information Science and Electrical Engineering, Kyushu University, Fukuoka 819-0395, Japan

**Keywords:** odometry, 6-DOF, IMU, neural networks

## Abstract

This paper presents an end-to-end learning framework for performing 6-DOF odometry by using only inertial data obtained from a low-cost IMU. The proposed inertial odometry method allows leveraging inertial sensors that are widely available on mobile platforms for estimating their 3D trajectories. For this purpose, neural networks based on convolutional layers combined with a two-layer stacked bidirectional LSTM are explored from the following three aspects. First, two 6-DOF relative pose representations are investigated: one based on a vector in the spherical coordinate system, and the other based on both a translation vector and an unit quaternion. Second, the loss function in the network is designed with the combination of several 6-DOF pose distance metrics: mean squared error, translation mean absolute error, quaternion multiplicative error and quaternion inner product. Third, a multi-task learning framework is integrated to automatically balance the weights of multiple metrics. In the evaluation, qualitative and quantitative analyses were conducted with publicly-available inertial odometry datasets. The best combination of the relative pose representation and the loss function was the translation and quaternion together with the translation mean absolute error and quaternion multiplicative error, which obtained more accurate results with respect to state-of-the-art inertial odometry techniques.

## 1. Introduction

Odometry is a process to compute relative sensor pose changes between two sequential moments. This is generally essential for various applications that need to track target device poses in a 3D unknown environment. Especially, estimating a 6 degrees of freedom (DOF) pose containing both a 3D position and a 3D orientation is crucial for the pose tracking of a drone in Robotics and Automation [1] and the registration of 3D annotations in Augmented Reality [2].

Recent approaches on the 6-DOF odometry are mainly based on the use of cameras, referred to as visual odometry [3]. Inertial measurement unit (IMU) is further integrated so that the odometry estimation can be stabilized even under fast motion [1]. The advantage of camera based approaches is the higher accuracy of estimated 6-DOF poses owing to less drift error, compared with other positioning sensors. However, the accuracy is largely degraded by appearance changes caused by moving objects and illumination. Also, the computational cost is rather higher due to the feature extraction and matching on hundred thousands of pixels.

It is useful if the odometry can be achieved by using low dimensional inertial data from an IMU in terms of the computational efficiency and the robustness to the surrounding changes. However, the naive approach based on the double integration of acceleration causes a critical drift error. The main difficulty of such inertial dead reckoning is to simultaneously estimate noise, bias, and gravity direction in the acceleration reading for accurately computing the linear acceleration caused by sensor motion only. To solve this problem, machine learning based approaches have recently been introduced. For instance, the velocity of the IMU attached on a human body is regressed by using the velocity from visual odometry as a ground truth for the training process [4]. Also, the location and direction on a 2D floor map can be regressed with a deep learning framework [5]. Estimating magnitude of translation and rotation changes using deep neural networks can also be performed [6]. However, end-to-end learning based 6-DOF odometry regression has not been achieved yet.

In this paper, we propose a 6-DOF odometry method only with an IMU based on a neural network trained with end-to-end learning. The network architecture follows a convolutional neural network (CNN) combined with a two-layer stacked bidirectional long short-term memory (LSTM). Especially, the network is designed from the following three aspects: 6-DOF relative pose representations, 6-DOF pose distance metrics, and the use of multi-task learning for balancing the metrics. First, a 6-DOF relative pose expressed in the spherical coordinate system, or a 3D translation vector and an unit quaternion are used. Second, mean squared error (MSE), translation mean absolute error (MAE), quaternion multiplicative error and quaternion inner product as pose distances are applied to the loss function in the network. Third, a multi-task learning that automatically balances the different metrics is integrated to handle the metrics for translation and rotation. Qualitative and quantitative evaluations with publicly available inertial odometry datasets showed that the combination of translation and quaternion based relative pose representation with translation MAE and quaternion multiplicative error based loss functions obtained the most accurate 6-DOF inertial odometry results, being superior to recent inertial odometry methods.

## 2. Related Work

The strapdown inertial navigation system (SINS) can be considered as the most straightforward approach to perform the 6-DOF odometry only with an IMU [7]. It works by naively double-integrating linear accelerations in the inertial reference frame of the system. However, the Micro Electro Mechanical System (MEMS) based inertial sensors installed on current robots, vehicles and mobile devices present a large amount of noise in the sensor data readings. This factor makes SINS quickly accumulate positional error over time, making them unsuitable for the odometry tasks.

By constraining the odometry problem to human motion estimation, pedestrian dead reckoning (PDR) systems can estimate the pedestrian trajectory on a 2D map by performing orientation update, step detection and step length estimation based solely on an IMU [8]. Generally, the method is optimized according to the location of a body part for mounting an IMU, such as foot, wrist, head and chest. These systems provide good trajectory estimation dedicated to pedestrians. However, they are generally limited to 2D pedestrian odometry, not being applicable to 6-DOF general cases.

One way to avoid the error accumulation in the IMU based 6-DOF odometry is to use it in conjunction with a monocular camera. There are several 6-DOF visual-inertial odometry (VIO) methods available, such as VINS-MONO [9] and OKVIS [10], and their performance was summarized in [1]. Owing to the recent advance of deep learning, end-to-end approaches were proposed in [11,12], which employ a neural network to fuse visual and inertial data. PIVO [13] is considered an inertial-visual odometry technique because a higher emphasis is given to the use of an IMU in order to be more robust to lack of discriminative features in the images. This is accomplished by using an Extended Kalman Filter (EKF) estimation. Nevertheless, making use of visual odometry causes an increase of energy consumption and processing demands, and a decrease of the accuracy under ill-conditioned surroundings.

Recently, machine learning techniques have been applied to the purely inertial odometry problem, being able to obtain superior results, compared with SINS and PDR. RIDI [4] estimated phone motion attached on a human body by first using support-vector machine to classify phone attachment location such as leg, bag, hand or body, and then employing support-vector regression trained for the given location to predict the device velocity. Such regressed velocity is finally used to correct accelerations on a 2D map, which are then double integrated to compute the odometry. This method is basically classified into PDR. IONet [5] employed LSTM to obtain displacements in polar coordinates from the IMU data. However, this method also focused only on estimating 2D planar trajectory as well as PDR, which is 3-DOF odometry.

The handheld INS described in [14] is able to perform purely inertial 6-DOF odometry on phones using EKF. It was later extended to constrain the velocity by using a CNN in [15]. Nevertheless, this approach is not solved by an end-to-end manner, needing to take additional processing for the device velocity, which can inherently be handled by our proposed method. AbolDeepIO [6] used LSTM to estimate the magnitude of 3D translation and rotation changes, but it is not able to predict trajectories from such outputs. To the best of our knowledge, there are no end-to-end methods for the 6-DOF inertial odometry with a low-cost IMU in the literature.

## 3. Materials and Methods

The proposed solution for the 6-DOF odometry with an IMU takes a sequence of gyroscope and accelerometer readings as input, and outputs a relative pose between two sequential moments. By successively performing this operation over time, a 3D trajectory can be estimated. Given an initial position and orientation, the computed pose changes are incrementally composed to finally obtain the pose in the reference coordinate system. Owing to an end-to-end learning framework, our solution implicitly handles inertial sensor bias and noise.

### 3.1. Network Architecture

**Figure 1:**
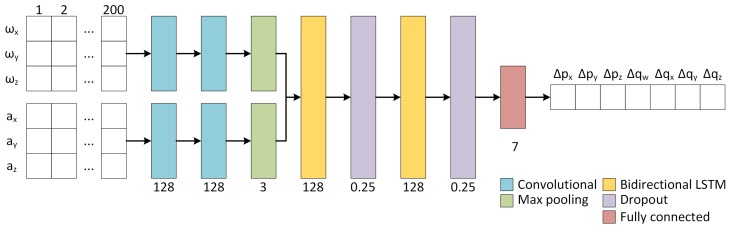
Network architecture for 6-DOF inertial odometry. The number of features is shown below the convolutional layers, the pool size is shown below the max pooling layers, the number of units is shown below LSTM and fully connected layers, and the dropout rate is shown below the dropout layers. The example output uses the 3D translation vector and unit quaternion based relative pose representation.

As illustrated in Figure 1, our network is based on CNN combined with LSTM, which is a type of recurrent neural network (RNN) that is highly suitable to problems that involve sequence processing [16]. As similar to [4,5,15], the input is the inertial data in a window of 200 frames, containing 3-axis angular velocity ω and 3-axis acceleration a. Gyroscope and accelerometer data are first processed separately by 1D convolutional layers of 128 features with a kernel size of 11. After two convolutional layers, a max pooling layer of size 3 is used. The output of these layers is concatenated and fed to LSTM layers with 128 units. Especially, a bidirectional LSTM is used, as in [5], so that both past 100 and future 100 IMU readings have an influence on the regressed relative pose. This is combined with a two-layer stacked LSTM model, in which a bidirectional LSTM outputs a full sequence that is the input of a second bidirectional LSTM. A dropout layer with a rate of 25% is also added after each LSTM layer in order to avoid overfitting. Finally, a fully connected layer generates the output of a relative pose.

**Figure 2:**
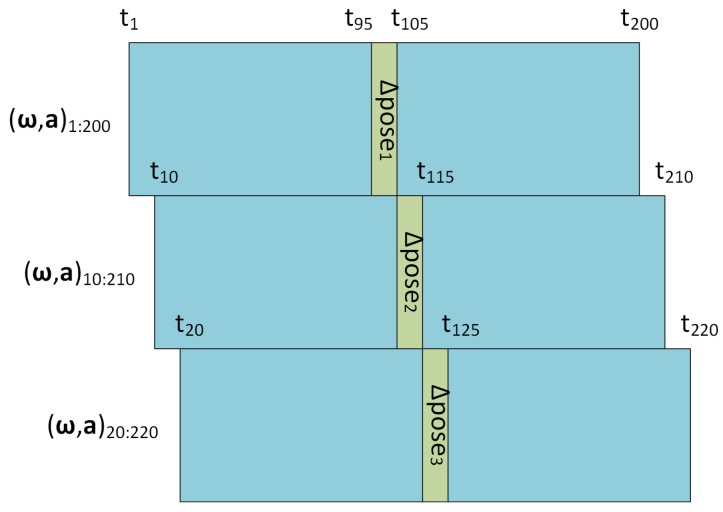
Input and output on time-axis. IMU data windows are overlapped over time (blue), and both past and future frames are used when computing the relative pose at each Δ pose moment (green).

As in [4,5], consecutive IMU reading windows have a stride of 10 frames. In this case, a new relative pose is computed every 10 frames, as illustrated in Figure 2. Given an IMU reading window of 200 frames, the relative pose to be regressed from this window is the one occurred between frames #95 and #105. This allows the correct composition of relative poses for estimating a trajectory with a slight delay according to the sampling rate, while also enabling the bidirectional LSTM approach to greatly benefit from both previous and future frames. It should be noted that it would be possible not to have such delay without exploiting future frames in exchange for a loss in accuracy.

### 3.2. 6-DOF Relative Pose Representation

There are several approaches to represent a 6-DOF relative pose. One approach is to simply extend the polar coordinate system proposed in [5] to the 3D space by using the spherical coordinate system. In the spherical coordinate system, the relative pose is represented by the traveled distance Δl, the inclination change Δθ and the heading change Δψ. Given a previous position $\left(x_{t-1},y_{t-1},z_{t-1}\right)$, a previous inclination $\theta_{t-1}$ and a previous heading $\psi_{t-1}$, the current location $\left(x_{t},y_{t},z_{t}\right)$ after a pose change (Δl,Δθ,Δψ) is obtained by

(1) $$\left\{\begin{array}{c} x_{t}=x_{t-1}+\Delta l\cdot \sin\left(\theta_{t-1}+\Delta \theta \right)\cdot \cos\left(\psi_{t-1}+\Delta \psi \right)\\ y_{t}=y_{t-1}+\Delta l\cdot \sin\left(\theta_{t-1}+\Delta \theta \right)\cdot \sin\left(\psi_{t-1}+\Delta \psi \right)\\ z_{t}=z_{t-1}+\Delta l\cdot \cos\left(\theta_{t-1}+\Delta \theta \right) \end{array}\right..$$

This allows obtaining a correct trajectory. However, one drawback is that the orientation will only be consistent when forward motion occurs. For example, if the system moves backwards or sideways with no change in the orientation, this will be interpreted as a forward movement together with a change of orientation in the backward or sideway direction.

Another approach is to use a 3D translation vector Δp and a unit quaternion Δq. This representation correctly handles the orientation when dealing with motions in any direction. From a previous position $p_{t-1}$ and orientation $q_{t-1}$, the current position $p_{t}$ and orientation $q_{t}$ after applying a pose change (Δp,Δq) is given by

(2) $$\left\{\begin{array}{c} p_{t}=p_{t-1}+R\left(q_{t-1}\right)\Delta p\\ q_{t}=q_{t-1}⊗\Delta q \end{array}\right.,$$

where R(q) is the rotation matrix for q, and ⊗ is the Hamilton product. The quaternions predicted by the neural network need to be normalized in order to ensure that they have unit length. In our experiments, we noted that the predicted quaternions before normalization have an average norm of 4.91, justifying their explicit correction.

### 3.3. 6-DOF Pose Distance Metric

A straightforward approach to estimate the difference between ground truth and predicted poses is to compute their MSE. This is done when using the spherical coordinates representation, namely $L_{MSE}$ loss function. However, MSE is an algebraic rather than a geometric distance function.

For the 6-DOF pose representation that employs quaternions, loss functions more related to the actual geometric difference between the ground truth pose (p,q) and the predicted pose $\left(\hat{p},\hat{q}\right)$ can be defined. Therefore, one alternative is to use the absolute value of the 3D pose graph SLAM error metric described in [17]. In this case, the corresponding loss function is $L_{TMAE}+L_{QME}$, so that:

(3) $$\left\{\begin{array}{c} L_{TMAE}={∥\hat{p}-p∥}_{1}\\ L_{QME}=2\cdot {∥imag(\hat{q}⊗q^{*})∥}_{1} \end{array}\right.,$$

where imag(q) returns the imaginary part of q, and $q^{*}$ is the complex conjugate of q. It is worth noting that $\hat{q}$ is a normalized quaternion.

Another possibility is to replace the quaternion multiplicative error $L_{QME}$ by a metric based on the inner product of unit quaternions, as discussed in [18]. Then, the loss function becomes $L_{TMAE}+L_{QIP}$, with

(4) $$\left\{\begin{array}{c} L_{TMAE}={∥\hat{p}-p∥}_{1}\\ L_{QIP}=1-\left|\hat{q}\cdot q\right| \end{array}\right..$$

### 3.4. Multi-Task Learning for Metric Balancing

The most straightforward way to compute the loss for the 6-DOF odometry problem is to assume a uniform weighting of the losses for each output type such as rotation and translation. However, the weight largely affects the results because these outputs have different nature and scale [19]. Therefore, estimating each of them is required to be treated as a separate task. For this problem, we propose to apply a multi-task learning framework to find a better weighting for the losses of these *n* tasks that share a common knowledge.

Inspired by [20], we aim to maximize the log Gaussian likelihood of the model, which is equivalent to minimizing the following final loss function:

(5) $$L_{MTL}=\sum_{i=1}^{n}\exp\left(-\log\sigma_{i}^{2}\right)L_{i}+\log\sigma_{i}^{2},$$

where $\sigma_{i}^{2}$ and $L_{i}$ are the variance and the loss function for the *i*-th task, respectively. The log variance values act as weights to the individual losses of each task. The goal of the training procedure is then to learn the $\log\sigma_{i}^{2}$ that minimizes $L_{MTL}$ from the ground truth data. As stated in [20], predicting the log variance is more numerically stable than regressing the variance, since for example divisions by zero are avoided.

In our network, a new multi-loss layer responsible for computing $L_{MTL}$ is added after inertial odometry layers in Figure 1. This means that the multi-loss layer takes each component of the 6-DOF pose predicted by the odometry layers, as input. The trainable weights of the multi-loss layer are the log variance values $\log\sigma_{i}^{2}$, each one associated to a layer input. All these weights are initially set to 0. Based on the predicted 6-DOF pose and the current values of $\log\sigma_{i}^{2}$, the layer computes $L_{MTL}$, and sets it as the final loss to be optimized. After the training, at prediction time, only the 6-DOF inertial odometry layers with their corresponding learned weights are used to regress the relative poses.

**Figure 3:**
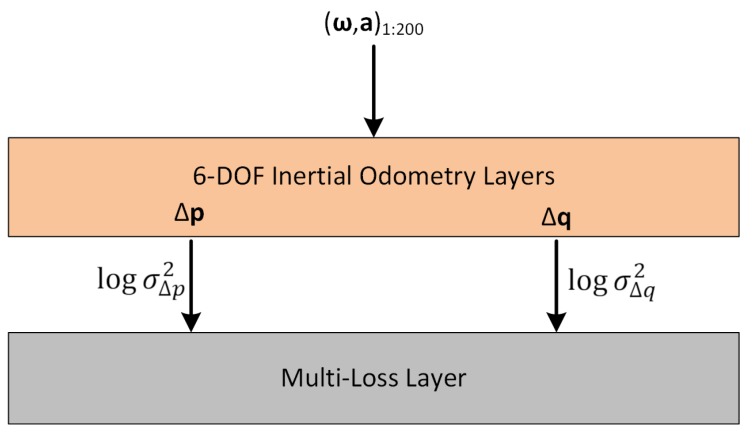
Network architecture with multi-task learning for the translation vector with quaternion. The multi-loss layer allows learning the weights (log variances) associated to each of two tasks (translation and orientation change estimation).

Figure 3 depicts the multi-task learning approach when the translation vector with quaternion representation is used. For example, if the quaternion multiplicative error is chosen as the orientation loss, one could use the following individual loss functions: $L_{1}=L_{TMAE}$ and $L_{2}=L_{QME}$.

### 3.5. Datasets

Experiments were performed using sequences obtained with a handheld smartphone and a micro aerial vehicle (MAV). For the handheld case, sequences from the Oxford Inertial Odometry Dataset (OxIOD) [21] were used. For the MAV scenario, the evaluation was performed using the sequences from the EuRoC MAV dataset [22].

OxIOD provides angular velocity and linear acceleration data recorded with phones at a sampling rate of 100 Hz while moving around the environment under different conditions. It also contains precise and synchronized ground truth 6-DOF poses. The data collected from user #1 holding an iPhone 7 Plus by hand while normally walking in a room was used, with a total of 24 sequences, a recording time of approximately 2 h and 27 min and a walking distance of 7.193 km. Due to the presence of noise in the ground truth measurements, the initial 12 s and the final 3 s of each sequence were discarded. From this data, 17 sequences were randomly chosen for the training and the remaining sequences were used for the testing, as can be seen in Table 1. Training IMU data windows also have a stride of 10 frames, resulting in a total amount of 55,003 training samples. Excerpts of 20 s from the test sequences were employed for both qualitative and quantitative evaluations.

EuRoC MAV dataset provides angular velocity and raw acceleration data recorded with an AscTec Firefly MAV equipped with a ADIS16448 IMU at a sampling rate of 200 Hz. Precise and synchronized ground truth 6-DOF poses are also provided. The dataset contains 11 sequences, a recording time of approximately 23 min and a total trajectory length of 894 m. It was adopted the same train and test splits from [6], which is shown in Table 2, with 6 sequences for the training and 5 sequences for the testing. Considering the stride of 10 frames for training IMU reading windows, we have a total amount of 13,122 training samples.

In order to generate ground truth data when using spherical coordinates, given a location change (Δx,Δy,Δz) associated to an IMU data window, a previous inclination $\theta_{t-1}$ and a previous heading $\psi_{t-1}$, the corresponding relative pose (Δl,Δθ,Δψ) can be obtained by

(6) $$\left\{\begin{array}{c} \Delta l=\sqrt{{\Delta x}^{2}+{\Delta y}^{2}+{\Delta z}^{2}}\\ \Delta \theta =\arccos\frac{\Delta z}{\Delta l}-\theta_{t-1}\\ \Delta \psi =\arctan\frac{\Delta y}{\Delta x}-\psi_{t-1} \end{array}\right.,$$

where Δθ and Δψ are enforced to be within [−π,π]. When the translation vector and quaternion based representation is used, a relative pose (Δp,Δq) is computed from previous and current positions and orientations $p_{t-1}$, $q_{t-1}$, $p_{t}$, $q_{t}$ associated to a given IMU data window as follows:

(7) $$\left\{\begin{array}{c} \Delta p=R^{T}\left(q_{t-1}\right)\left(p_{t}-p_{t-1}\right)\\ \Delta q=q_{t-1}^{*}⊗q_{t} \end{array}\right..$$

Uniqueness of quaternion Δq is enforced by constraining it to the upper hemisphere of $S^{3}$.

### 3.6. Detail of Training

**Figure 4:**
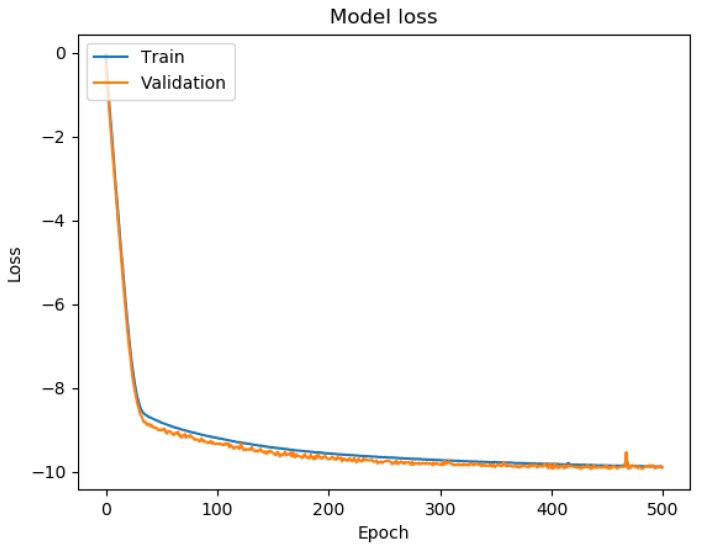
Training and validation loss of the proposed multi-task learning approach using translation vector with quaternion as relative pose representation and translation MAE with quaternion multiplicative error as individual task losses.

The Adam optimizer [23] was used with a learning rate of 0.0001. Keras (https://keras.io/) 2.2.4 together with TensorFlow (https://www.tensorflow.org/) 1.13.1 were employed. The training was done on a single NVIDIA GeForce GTX 1050 Ti GPU with a batch size of 32 samples. The neural network was trained for 500 epochs. 10% of the training data was used as validation data in the training. The model with best validation loss throughout training was chosen as the final one for the testing. Figure 4 shows an example of training and validation loss lines obtained with the proposed approach. The best validation loss was achieved in epoch #436, which justifies the choice of 500 epochs.

## 4. Results

Quantitative and qualitative evaluations were conducted in order to assess the effectiveness of the proposed 6-DOF inertial odometry solution.

### 4.1. OxIOD Handheld Qualitative Evaluation

Table 3 presents the variants of the proposed method that were evaluated using OxIOD handheld sequences. All of them use $L_{MTL}$ as the final weighted loss function.

**Figure 5:**
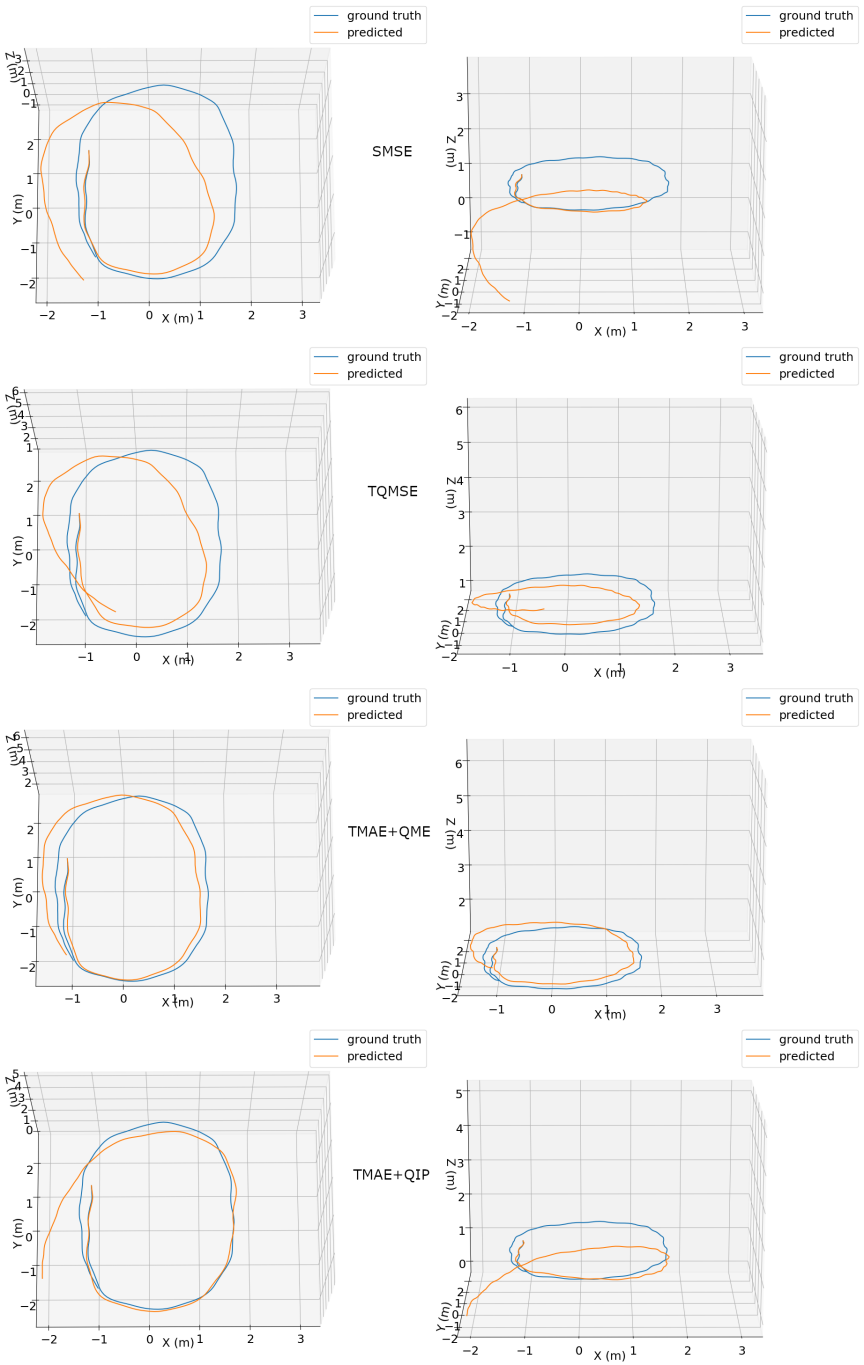
Top (**left column**) and side (**right column**) views of ground truth (blue) and predicted (orange) trajectories for an excerpt of the “handheld/data5/seq1” dataset using different configurations: SMSE (**first row**), TQMSE (**second row**), TMAE+QME (**third row**) and TMAE+QIP (**fourth row**).

**Figure 6:**
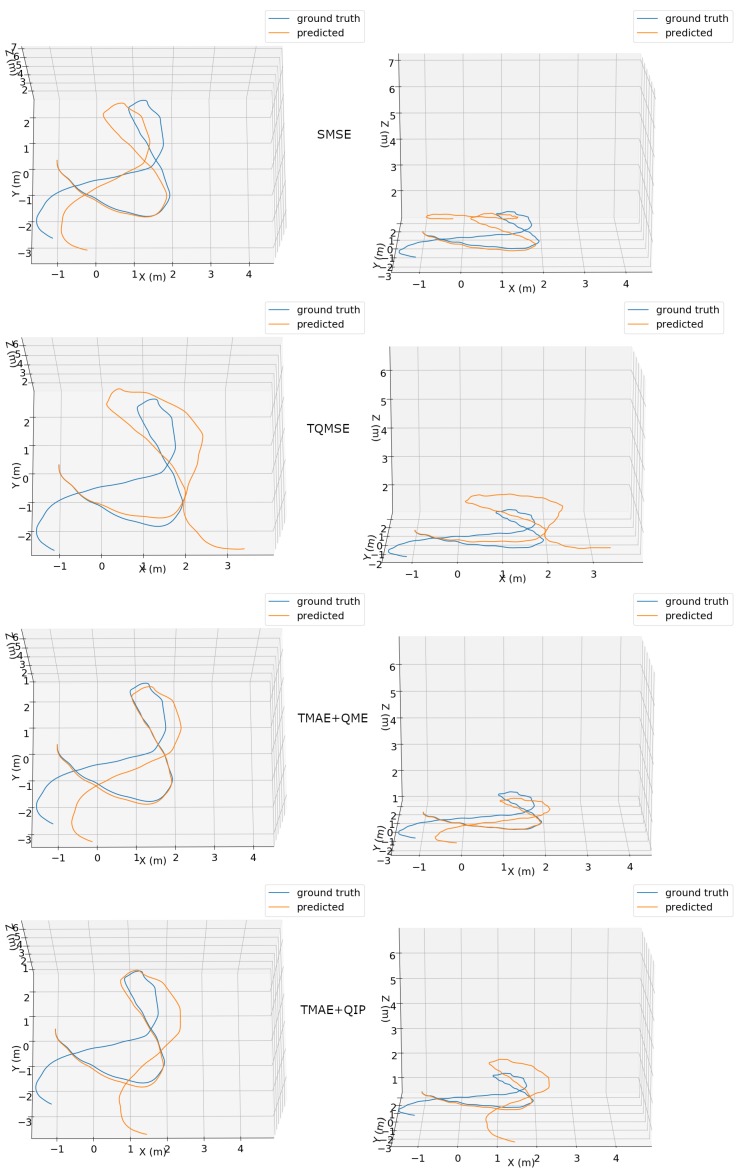
Top (**left column**) and side (**right column**) views of ground truth (blue) and predicted (orange) trajectories for an excerpt of the “handheld/data4/seq3” dataset using different configurations: SMSE (**first row**), TQMSE (**second row**), TMAE+QME (**third row**) and TMAE+QIP (**fourth row**).

Visual representations of the predicted 3D trajectories using our proposed method for two different sequences are presented in Figure 5 and Figure 6. Each plot shows aligned ground truth and predicted trajectories. The trajectories are shown in both top and side perspectives, allowing the assessment of 6-DOF pose estimation. The TMAE+QME configuration clearly obtained the most accurate trajectory, being able to precisely describe the circular-like path in Figure 5 and obtaining the best results with a more complex motion shown in Figure 6.

### 4.2. OxIOD Handheld Quantitative Evaluation

In order to quantitatively compare the different configurations in our proposed method, the root-mean-square error (RMSE) of the predicted trajectories was computed for the excerpts of all 7 test sequences considered. It should be noted that changes in both position and orientation affect the estimated trajectory. Table 4 lists the name of each sequence used together with the corresponding RMSE of the trajectory estimated by a given variant of the method. TMAE+QME was the best in most of the cases. The mean RMSE of TMAE+QME was nearly 60% of both TMAE+QIP and TQMSE and less than one third of the SMSE one.

The average 6-DOF relative pose prediction time for all configurations was ≈8 ms. Therefore, the technique is able to work in an interactive way.

### 4.3. EuRoC MAV Qualitative Evaluation

**Figure 7:**
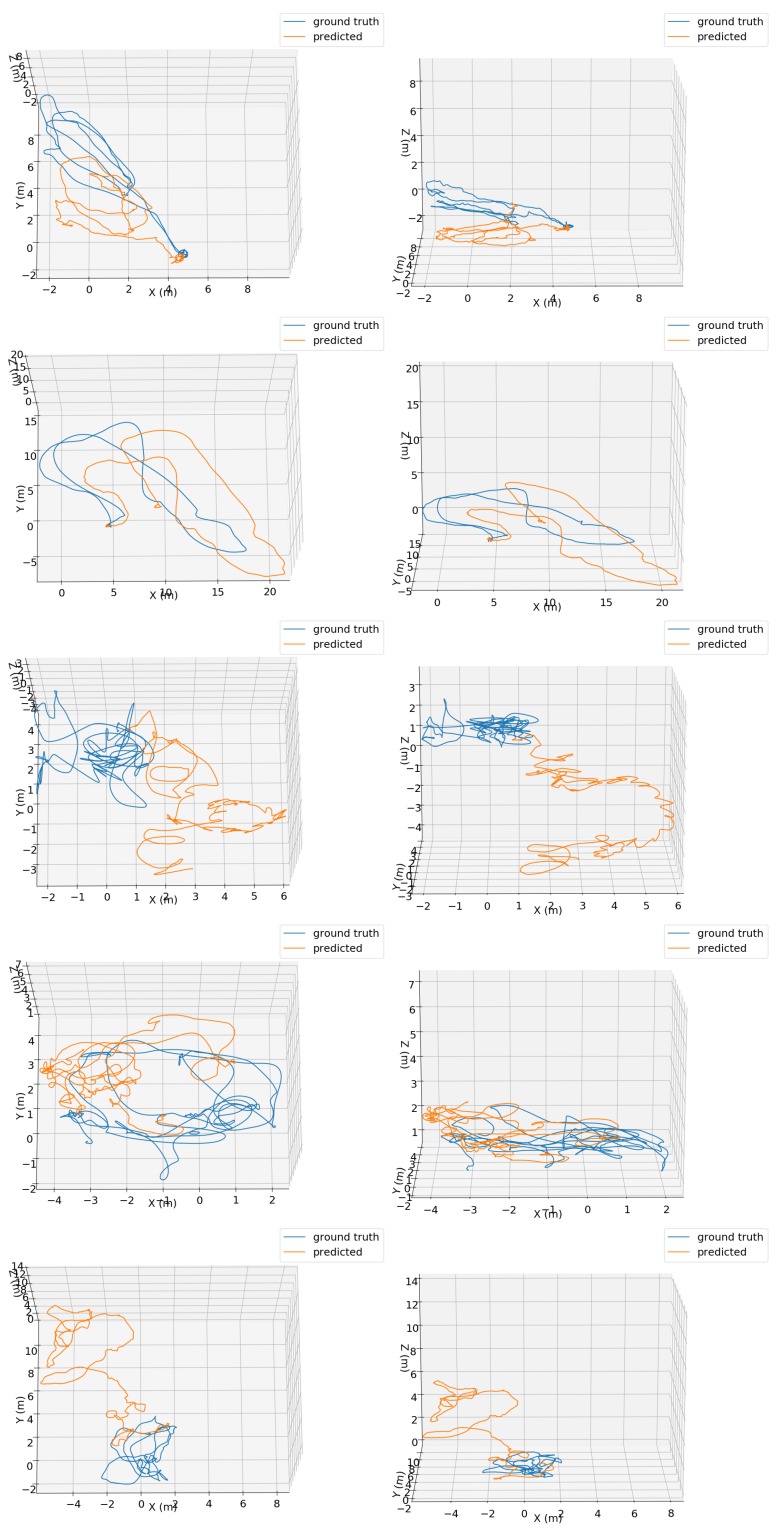
Top (**left column**) and side (**right column**) views of ground truth (blue) and predicted (orange) trajectories using the TMAE+QME configuration for different test sequences of the EuRoC MAV dataset: MH_02_easy (**first row**), MH_04_difficult (**second row**), V1_03_difficult (**third row**), V2_02_medium (**fourth row**) and V1_01_easy (**fifth row**).

Since TMAE+QME was the best one in the tests using the OxIOD handheld datasets, only this configuration was considered for the evaluations using the EuRoC MAV dataset. Top and side perspectives of predicted 3D trajectories for all EuROC MAV test sequences using our TMAE+QME method together with aligned ground truth trajectories are shown in Figure 7. One of the sequences is depicted in the Supplementary Video. Our TMAE+QME method is able to obtain coherent trajectories, despite having some error accumulation.

### 4.4. EuRoC MAV Quantitative Evaluation

Table 5 shows the RMSE of the predicted trajectories obtained by our TMAE+QME method for the 5 test sequences from the EuRoC MAV dataset. Since these sequences are longer, with an average time of ≈2 min, there is more error accumulation, which explains the increased values with respect to the OxIOD handheld experiments.

We also compared MAE and RMSE for magnitude of translation changes over windows of 10 frames with the values reported in [6]. Our TMAE+QME results are compared with SINS, IONet [5], VINet [11], and AbolDeepIO2 [6] in Table 6. Our TMAE+QME method outperformed competing techniques in almost all of the cases. The overall MAE and RMSE of our TMAE+QME approach were ≈70% of the best competing method, which was AbolDeepIO2. The magnitude of rotation changes over windows of frames are also reported in [6]. Nevertheless, it was not possible to compare our TMAE+QME results with them, since we use unit quaternions for representing orientation, so their magnitude is always equals to one.

### 4.5. Limitation

**Figure 8:**
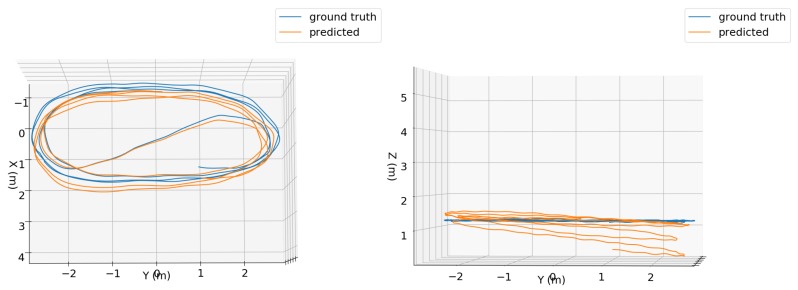
Top (**first column**) and side (**second column**) views of ground truth (blue) and predicted (orange) trajectories for 1 minute of the “handheld/data4/seq1” sequence using the TMAE+QME configuration.

Different from [14,15], no position fixes nor manual loop closures were used by our method in any of the experiments. Due to this, error accumulation occurred in the estimated trajectories when handling some long sequences, especially in the Z position coordinates. Figure 8 and the Supplementary Video illustrates this, where the predicted trajectory using the TMAE+QME configuration for a 1-minute sequence is compared with the ground truth. It is possible to note an increased error in the position estimates along the Z axis. We were not able to establish a relationship between trial dynamics and observed errors. For example, in the EuRoC MAV dataset, MH_04_difficult and V1_03_difficult present fast motion and obtained the second and third worst errors, but the worst results were obtained with the V1_01_easy sequence, which exhibits slow motion. In addition, the tests with the V2_02_medium sequence, which presents fast motion, showed the best results. We should also have in mind that, since all the OxIOD handheld sequences used for training were collected by the same user and with the same device and motion model, problems may be experienced when testing with different users/devices/motion models.

## 5. Conclusions

It was presented an odometry technique that works in an end-to-end manner and is able to successfully provide 6-DOF relative pose estimates using solely noisy and biased inertial data obtained from a low-cost IMU. The proposed approach is based on convolutional layers combined with a two-layer stacked bidirectional LSTM deep learning model. In addition, a multi-task learning approach was adopted, which automatically finds the best weights for the individual losses associated to rotation and translation. The translation vector with unit quaternion 6-DOF based relative pose representation provided better predicted trajectories than the spherical coordinate ones in all the tests. Regarding the loss functions, best results were obtained when using translation MAE and quaternion multiplicative error, respectively. The conducted experiments showed that the proposed method was superior to state-of-the-art inertial odometry techniques.

As future work, we plan to tackle the error accumulation issue in some long sequences by performing a visual update to the 6-DOF inertial odometry, similar to PIVO [13]. However, this update does not need to happen at every camera frame. It can be done over frame batches or only when there is a sufficient level of certainty in the visual information. This would make the system to trust more on the purely inertial odometry in scenarios when there are not many reliable visual features. It would also allow to save processing time and energy consumption. Investigations will be performed regarding the use of traditional approaches for computing orientation such as [24] together with translation regression using deep learning to check if this would yield better results. Although OxIOD also provides magnetometer data, from our experience such information is less reliable due to noise caused by magnetic fields from electrical devices. Nevertheless, other strategies such as zero-velocity updates (ZUPTs) can be adopted to improve trajectory estimation when the system becomes stationary, as done in [14]. We also plan to perform domain adaptation using a generative adversarial network (GAN) in order to better handle data collected by different users and with different devices and motion models [25].

## Figures and Tables

**Table 1:** Train and test splits of OxIOD handheld sequences.

Training Data	Testing Data
data1/seq1	data1/seq2
data1/seq3	data1/seq5
data1/seq4	data1/seq6
data1/seq7	data3/seq1
data2/seq1	data4/seq1
data2/seq2	data4/seq3
data2/seq3	data5/seq1
data3/seq2	
data3/seq3	
data3/seq4	
data3/seq5	
data4/seq2	
data4/seq4	
data4/seq5	
data5/seq2	
data5/seq3	
data5/seq4	

**Table 2:** Train and test splits of EuRoC MAV dataset.

Training Data	Testing Data
MH_01_easy	MH_02_easy
MH_03_medium	MH_04_difficult
MH_05_difficult	V1_03_difficult
V1_02_medium	V2_02_medium
V2_01_easy	V1_01_easy
V2_03_difficult	

**Table 3:** Evaluation configuration for 6-DOF relative pose representation and individual task losses: spherical coordinates + mean squared error (SMSE), translation and quaternion + mean squared error (TQMSE), translation mean absolute error + quaternion multiplicative error (TMAE+QME) and translation mean absolute error + quaternion inner product (TMAE+QIP).

Configuration	6-DOF Relative Pose	Individual Task Losses
SMSE	(Δl,Δθ,Δψ)	$L_{MSE}$
TQMSE	(Δp,Δq)	$L_{MSE}$
TMAE+QME	(Δp,Δq)	$L_{TMAE}$, $L_{QME}$
TMAE+QIP	(Δp,Δq)	$L_{TMAE}$, $L_{QIP}$

**Table 4:** Comparison of trajectory RMSE in meters for excerpts of the test sequences from OxIOD.

Sequence	SMSE	TQMSE	TMAE+QME	TMAE+QIP
data1/seq2	1.832	**0.619**	0.681	0.632
data1/seq5	2.394	0.667	**0.575**	1.172
data1/seq6	1.405	0.334	0.615	**0.282**
data3/seq1	1.453	0.763	**0.353**	0.470
data4/seq1	1.295	0.894	**0.370**	1.006
data4/seq3	0.847	1.760	**0.518**	1.156
data5/seq1	1.328	0.682	**0.265**	0.825
Mean	1.508	0.817	**0.482**	0.792

**Table 5:** Trajectory RMSE in meters for the test sequences from EuRoC MAV dataset.

Sequence	TMAE+QME
MH_02_easy	3.307
MH_04_difficult	5.199
V1_03_difficult	5.329
V2_02_medium	2.897
V1_01_easy	8.166
Mean	4.980

**Table 6:** Comparison of MAE/RMSE for magnitude of translation change over 10 IMU frames using the test sequences from EuRoC MAV dataset.

Sequence	SINS	IONet	VINet	AbolDeepIO2	TMAE+QME
MH_02_easy	0.0212/0.0251	0.0115/0.0140	0.0116/0.0143	0.0095/0.0125	**0.0077**/**0.0101**
MH_04_difficult	0.0437/0.0544	0.0274/0.0350	0.0293/0.0376	0.0199/0.0265	**0.0092**/**0.0127**
V1_03_difficult	0.0346/0.0399	0.0167/0.0218	0.0172/0.0222	0.0137/**0.0176**	**0.0130**/0.0177
V2_02_medium	0.0337/0.0387	0.0159/0.0199	0.0166/0.0209	0.0158/0.0202	**0.0131**/**0.0170**
V1_01_easy	0.0172/0.0198	0.0137/0.0169	0.0112/0.0135	0.0122/0.0150	**0.0075**/**0.0099**
All	0.0286/0.0358	0.0164/0.0217	0.0163/0.0221	0.0138/0.0183	**0.0098**/**0.0135**

## Supplementary Materials

The following are available online at https://www.mdpi.com/1424-8220/19/17/3777/s1.

## References

[1] Delmerico J., Scaramuzza D. A Benchmark Comparison of Monocular Visual-Inertial Odometry Algorithms for Flying Robots. Proceedings of the 2018 IEEE International Conference on Robotics and Automation.

[2] Marchand E., Uchiyama H., Spindler F. (2016). Pose estimation for augmented reality: A hands-on survey. IEEE Trans. Vis. Comput. Graph..

[3] Scaramuzza D., Fraundorfer F. (2011). Visual odometry [tutorial]. IEEE Robot. Autom. Mag..

[4] Yan H., Shan Q., Furukawa Y. RIDI: Robust IMU double integration. Proceedings of the European Conference on Computer Vision.

[5] Chen C., Lu C.X., Markham A., Trigoni N. IONet: Learning to Cure the Curse of Drift in Inertial Odometry. Proceedings of the Thirty-Second AAAI Conference on Artificial Intelligence.

[6] Esfahani M.A., Wang H., Wu K., Yuan S. (2019). AbolDeepIO: A Novel Deep Inertial Odometry Network for Autonomous Vehicles. IEEE Trans. Intell. Transp. Syst..

[7] Titterton D., Weston J.L., Weston J. (2004). Strapdown Inertial Navigation Technology.

[8] Lu C., Uchiyama H., Thomas D., Shimada A., Taniguchi R.-i. (2019). Indoor Positioning System Based on Chest-Mounted IMU. Sensors.

[9] Qin T., Li P., Shen S. (2018). VINS-MONO: A robust and versatile monocular visual-inertial state estimator. IEEE Trans. Robot..

[10] Leutenegger S., Lynen S., Bosse M., Siegwart R., Furgale P. (2015). Keyframe-based visual–inertial odometry using nonlinear optimization. Int. J. Robot. Res..

[11] Clark R., Wang S., Wen H., Markham A., Trigoni N. VINet: Visual-Inertial Odometry as a Sequence-to-Sequence Learning Problem. Proceedings of the AAAI.

[12] Chen C., Rosa S., Miao Y., Lu C.X., Wu W., Markham A., Trigoni N. Selective Sensor Fusion for Neural Visual Inertial Odometry. Proceedings of the Computer Vision and Pattern Recognition (CVPR-19).

[13] Solin A., Cortes S., Rahtu E., Kannala J. PIVO: Probabilistic inertial-visual odometry for occlusion-robust navigation. Proceedings of the 2018 IEEE Winter Conference on Applications of Computer Vision.

[14] Solin A., Cortés S., Rahtu E., Kannala J. Inertial odometry on handheld smartphones. Proceedings of the FUSION 2018 International Conference.

[15] Cortés S., Solin A., Kannala J. Deep learning based speed estimation for constraining strapdown inertial navigation on smartphones. Proceedings of the IEEE 28th International Workshop on Machine Learning for Signal Processing.

[16] Jozefowicz R., Zaremba W., Sutskever I. An empirical exploration of recurrent network architectures. Proceedings of the 32nd International Conference on International Conference on Machine Learning.

[17] Ceres Solver. http://ceres-solver.org.

[18] Huynh D.Q. (2009). Metrics for 3D rotations: Comparison and analysis. J. Math. Imaging Vis..

[19] Kendall A., Grimes M., Cipolla R. Posenet: A convolutional network for real-time 6-dof camera relocalization. Proceedings of the IEEE International Conference on Computer Vision.

[20] Kendall A., Gal Y., Cipolla R. Multi-task learning using uncertainty to weigh losses for scene geometry and semantics. Proceedings of the IEEE Conference on Computer Vision and Pattern Recognition.

[21] Chen C., Zhao P., Lu C.X., Wang W., Markham A., Trigoni N. (2018). OxIOD: The Dataset for Deep Inertial Odometry. arXiv Preprint.

[22] Burri M., Nikolic J., Gohl P., Schneider T., Rehder J., Omari S., Achtelik M.W., Siegwart R. (2016). The EuRoC micro aerial vehicle datasets. Int. J. Robot. Res..

[23] Kingma D.P., Ba J.L. Adam: A method for stochastic optimization. Proceedings of the 3rd International Conference for Learning Representations.

[24] Madgwick S.O., Harrison A.J., Vaidyanathan R. Estimation of IMU and MARG orientation using a gradient descent algorithm. Proceedings of the 2011 IEEE International Conference on Rehabilitation Robotics.

[25] Chen C., Miao Y., Lu C.X., Xie L., Blunsom P., Markham A., Trigoni N. (2019). MotionTransformer: Transferring Neural Inertial Tracking between Domains. Proc. AAAI Conf. Artif. Intell..

